# Effect of Industrial Robots on Employment in China: An Industry Level Analysis

**DOI:** 10.1155/2022/2267237

**Published:** 2022-07-18

**Authors:** Yantong Zhao, Rusmawati Said, Normaz Wana Ismail, Hanny Zurina Hamzah

**Affiliations:** ^1^School of Business and Economics, University Putra Malaysia, 43400 Serdang, Selangor, Malaysia; ^2^College of Confucian Business, Jining University, Qufu 273155, Shandong, China

## Abstract

China has been the world's largest market for industrial robots since 2013. Industrial robots improve accuracy, safety, and efficiency in industrial production but have a substantial impact on the labor market. This investigation uses the task-based model to explore the relationship between industrial robots and employment across industries. This study uses industrial robot data from the International Federation of Robotics and employment data from the China Statistical Yearbook from 2010 to 2019 to examine robot applications' influencing mechanisms on labor demand in different industries in China. The results show a significant positive correlation between robots' exposure and labor demand for IT, health and social services, science research and technical services, and management of water conservancy and environmental industries. Based on the results, the use of robots promotes high-skilled talent employment and some third-sector employment, like education, food and beverages, utilities, household appliances, and transport. However, multiple regression analysis reveals that the use of robots has reduced employment in traditional industries such as agriculture and mining.

## 1. Introduction

The issue of the potential impact of robots on the labor market has received considerable attention. The popularity of artificial intelligence and new robot designs has significantly influenced both the economy and society. At the beginning of the 20th century, the famous economist, John Maynard Keynes, predicted that humankind would have a new employment challenge, that is, “technological unemployment” [1]. Rapid progress in the fields of artificial intelligence and robotics has begun to solve major problems in terms of productivity and the acceleration of the replacement of labor by robots. This introduces tremendous challenges having to do with the human workforce [2]. According to application, robots can be classified as repetitive-tasks robots, medical robots, guarding robots, domestic-purpose robots, astronomical robots, entertainment robots, mining robots, defense and military robots, agriculture robots, and remote-areas robots [3]. A recent study by the McKinsey Global Institute (MGI) pointed out that robots will replace 400 to 800 million people in the world by 2030. In the rapid development of automation, up to 31% of working hours in China will be automated [4]. To what extent will robots replace human workers in the future? Is the rapid development of artificial intelligence and robotics responsible for introducing positive opportunities or challenges? This set of questions has become a global issue that cannot be ignored.

Since 2015, artificial intelligence has seen explosive growth in China, attracting significant attention from all social sectors. In September 2017, the government released a document entitled, “New Generation Artificial Intelligence Development Plan.” This plan urges China to seize the global command of artificial intelligence by the year 2030 and incubate 10 trillion Yuan in industrial output. As an essential component of artificial intelligence, robot applications have become a critical fulcrum for transforming China from manufacturing to higher-quality economic development. More robots are utilized from the IFR (IFR, 2019) data than ever before. The operational stock of robots measures the number of robots currently deployed. The working stock of robots in the world rose from 1.02 million in 2009 to 2.73 million units in 2019 with a steady upward trend. China, Japan, the United States, South Korea, and Germany constitute the five major industrial robot markets. They account for about 70% of the global import and export in trade in robots and their installation. The data from the past ten years show that robot operations in China are in a state of continuous growth. At the same time, the United States, Germany, Japan, and South Korea have experienced zero or even negative growth in the rate of robot ownership in the past ten years. China has been the world's largest market for industrial robots since 2013. The number of robot installation was about 550 units in 1990. After ten years of slow augmentation, robot installations increased from 5525 units in 2009 to 156,000 units in 2017. In 2017 and 2018, China's total industrial robot installations accounted for 38% of the global total. In 2019, a total of 140,000 units were installed which is 9% less than that in 2018 but still higher than the total robots installed in Europe and the United States. With the acceleration of artificial intelligence development, robots have had a greater impact on labor force employment. Against this backdrop, it is necessary to systematically study the technical progress of robots and their effect on the labor market from the perspective of economic method and theory.

Both the government and private enterprise in China have expended a tremendous amount of money to promote and support technological innovation in terms of artificial intelligence and robots' development. The rise of human-machine collaborations has affected all areas of the modern world, from work to everyday life and beyond. However, it is difficult to answer the question of whether robots are responsible for creating or eliminating employment opportunities. More than 100,000 new industrial robots were installed in the United States industry (IFR, 2019), mainly in the automotive industry where the employment increased by 230,000 jobs from 2010 to 2015 [5]. Although, certain occupations may disappear in local communities, the total number of jobs may increase globally. In addition to changing the jobs quantity, the nature and characteristics of employment and labour are likely to change fundamentally. As one of the largest developing countries with a transitional economy, the rapid popularization of robotic technology will undoubtedly have a profound dual effect on human resource management in the market. Previous studies about the impact of robots on the labor market, however, are inconsistent. Most studies of these studies have only been conducted in developed countries [6–9]. Although a few studies have investigated the development of robotics and artificial intelligence and the corresponding economic impact on China [10–12], most studies have focused on the theoretical dimension or country, provincial, and firm levels [13–15]. A wide gap also exists between China's robotic development and its impact. Systematic empirical studies are lacking on the relationship between industrial robots and China's employment in different industries and skills. This investigation aims to explore the relationship between industrial robots and employment across industries. This study uses the International Federation of Robotics (IFR)'s industrial robot data and employment data from the China Statistical Yearbook to construct a penetration index for industrial robots in China. Thus, we examine the influence of robot deployment on labor demand in China. We also analyze the potential impact of robots on economic outcomes, which may provide the Chinese government with guidance for economic growth and employment in the future.

The paper organization is as follows: the next section reviews the existing theoretical and empirical literature on industrial robots' impact on economic and labor markets.  Section 3 describes research methods, with a particular focus on the theoretical framework, data description, and the empirical model. The last section represents a summary of the research.

## 2. Literature Review

With the rapid development of artificial intelligence technology, a new era of technological revolution and industrial transformation is gradually taking shape. It is an era in which robots are changing manufacturing processes and life patterns [16]. The rapid development of the international robot market has become an important phenomenon that cannot be ignored in terms of its consequences for economic life. This section reviews the existing theoretical and empirical literature on the impact of industrial robots on the economy and labor market.

Some scholars believe that technological innovation leads to social progress and reduces the importance of human resources in the production sector, which increases the unemployment rate [2, 17, 18]. In examining this proposition, Susskind developed a task-based model. He argues that using intelligent machines can reduce relative wages and the income share of the labor force, while leading to a high unemployment rate [17]. Frey and Osborne developed a model based on the Gaussian process to classify more than 700 detailed occupations in the United States according to their susceptibility to automation based on data from the United States Department of Labor's Occupational Classification Database. The results of this line of investigation show that about half of jobs in the United States will be challenged by automation in the next decades [2]. Acemoglu and Restrepo [18] examine the competition between human labor and robots in terms of different production tasks. They conclude that the use of robots will reduce employment and lower the laborers' salaries. Their analysis is based on the usage of industrial machines, employment, and wage changes to return in the United States (IFR). According to the results of each additional robot in every thousand workers, the employed population ratio decreases by 0.18%∼0.34%, and the wage decreases by 0.25%∼0.5%. This negative effect Indicates that the application of robots has a significant negative impact on the employment and wage in the commuting field [18].

Many scholars also support the view that innovation positively affects employment [19–21]. Bloom et al. estimate that, due to the extensive integration of artificial intelligence technology into daily life, between 2010 and 2030, the world will develop 734 million new jobs [19]. Acemoglu and Restrepo [20] argue that, from the standpoint of the development history of science and technology, in the long run, technological progress will lead to the development of new employment opportunities. In this respect, the compensation effect produced by new jobs can offset the substitution effect caused by automation [20]. Similarly, Gregory et al. [21] examined data from 27 European countries from 1999 to 2010. They found that conventional forms of technological change led capital to replace labor in the production process, reducing employment by about 9.6 million jobs. In comparison, the spillover effect on product demand brought about by technological progress led to an increase of nearly 21 million jobs. On the whole, technological progress has had a net positive impact on employment in the European labor force [21].

A series of empirical studies have shown that unskilled labor and capital are substitutes, while skilled labor and capital are complementary [22–25]. For example, when machine prices fall, firms reduce the use of unskilled labor. In contrast, when the prices of machinery fall, manufacturers increase the use of equipment, and the demand for skilled labor increases because equipment operation requires skilled labor. According to the study, a 10% decrease in equipment prices leads to a 5% decrease in the use of unskilled labor and a 5% increase in the use of skilled labor [26]. This finding is widely known as the capital–skill complementary hypothesis, which has several important policy implications. For example, the hypothesis suggests that technological advances, such as the rapid drop in the cost of computing in recent decades, may significantly influence income inequality. This effect exists because technological progress causes an increase in demand for skilled labor and a decrease in demand for unskilled labor.

The impact of technological progress on the employment of laborers with different skills shows that highly skilled laborers can quickly master and adapt to new technologies; and, in this sense, skilled labor has a complementary relationship with new technologies. However, unskilled labor is limited by its level of human capital since unskilled laborer are unable to master new technologies quickly. For this reason, its risks are replaced by new technologies. As with the progress of traditional technology, artificial intelligence will increase the demand for and employment opportunities for skilled labor. This will create a substitution effect for unskilled labor [27–29]. In this respect, Acemoglu and Restrepo [20] point out that artificial intelligence and robot learning have enabled robots to make breakthroughs in analysis, problem-solving, and the performance of complex and unconventional tasks. It is also possible that robots can do the work of highly skilled workers instantly. By constructing a theoretical model, they found that the skilled labor force replaced by a high-skilled labor force might compete with low-skilled workers and be competent for other jobs. The employment creation effect of artificial intelligence on the labor force is thus concentrated in high- and low-skill positions, and the substitution effect is centered on medium-skill positions. This labor force has comparative advantages in terms of communication, service, innovation, and research and development (R&D) [20]. Artificial intelligence is complementary to the labor force in these sectors and can, in fact, create jobs.

The substitution and creation effects on labor caused by intelligent robots produce changes in labor supply and demand, and they can ultimately lead to changes in wage equilibrium. Previous studies have also explored the impact of robots on labor compensation from various perspectives. Some researchers conclude that intelligent robots can quickly replace workers in certain jobs and thereby reduce wages. The two-phase overlapping generations model proposed by Benzell et al. illustrates the idea that the operation of intelligent robots will cause labor's share of national income to decrease in the long run [30]. Similarly, DeCanio [31] uses the Houthakker model [32] to briefly evaluate the influence of intelligent robots on labor compensation in the United States [31]. Cabrales et al. demonstrated that the threat of robot replacement does not affect the efforts of workers [33]. According to DeCanio's results, if the job substitution elasticity of humans and robots exceeds 1.9, then the expansion of robots will induce salary reductions.

Several studies have examined the impact of robot adoption on the labor market in China [12–15]. Fan et al. examined the impact of rising labor costs on the adoption of industrial robots by Chinese companies. A 10% increase in the minimum wage between 2008 and 2012 increased the probability of a company adopting a robot by 0.11% points. Higher minimum wages have a significant impact on the adoption of robots for firms that are more productive, located in coastal areas, private, and skilled labor-intensive [13]. Tang et al. found that the adoption of robots and highly skilled workers are complementary. After adopting robots, companies hire more highly skilled and educated workers. Therefore, the adoption of robots has resulted in an employment skill bias among Chinese enterprises [12]. Du and Lin systematically investigated the impact of adopting industrial robots on total factor productivity in different regions according to the panel data of Chinese provinces from 2006 to 2019. The results showed a U-shaped relationship between the adoption of industrial robots and total factor productivity [14]. Fu et al. studied the labor markets of 74 economies using international panel datasets from 2004 to 2016. The study found that the adoption of industrial robots is associated with a significant increase in labor productivity and total employment in developed economies, whereas the impact is not significant in developing countries, where increased robot adoption is associated with a significant decline in the labor share of GDP. In both developed and developing countries, increased robot adoption is associated with a substantial increase in income inequality [15].

## 3. Research Methods

### 3.1. Theoretical Framework

For investigating the effect of industrial robots on employment, our research uses the task-based model with automation technology [7]. Firstly, we introduce the basic model, and then, in the second part, we replace the automated capital with robots to analyze the impact of robots on the labor market. In this way, we obtain the labor quantity under equilibrium conditions.

(I). Basic model: task-based model with automation technology
Assume that the economy produces only one good *Y*, accomplished by a series of tasks *y* (*i*), and the production function is as follows:
  (1) $$\begin{array}{c} Y={\left(\int_{N-1}^{N}y{\left(i\right)}^{\sigma -1/\sigma }\text{d}i\right)}^{\sigma /\sigma -1}, \end{array}$$
where *σ* is elasticity of substitution. An increase in *N* implies that a new complex task appears. An increase in *N* − 1 also means that the old task disappears.
Labor can produce any task. Capital is unable to produce complex new tasks. Assume that *I* ∈ [*N* − 1, *N*], when tasks *i* < *I*, it is feasible to produce with capital. If *i* > *I*, tasks *I* must be produced by labor. When *i<I*, the production function of task *i* is as follows:
  (2) $$\begin{array}{c} y\left(i\right)=k\left(i\right)+\gamma \left(i\right)\cdot l\left(i\right), \end{array}$$
where *γ* (*i*) is labor productivity, *l*(*i*) is labor, and *k*(*i*) is capital.
When *i* ≥ *I*, the production function of task *i* is as follows:
  (3) $$\begin{array}{c} y\left(i\right)=\gamma \left(i\right)\cdot l\left(i\right). \end{array}$$
The cost of each task is as follows:
  (4) $$\begin{array}{c} p\left(i\right)=\left\{\begin{array}{c} \min\left\{r,\frac{W}{\gamma \left(i\right)}\right\},\;i\leq I,\\ \frac{W}{\gamma \left(i\right)},\;i>I. \end{array}\right. \end{array}$$
Where *r* is capital rent and *W* is wage. From equations (2)–(4), we can get the following equation:
  (5) $$\begin{array}{c} y\left(i\right)=Y\cdot p{\left(i\right)}^{-\sigma },\\ y\left(i\right)=\left\{\begin{array}{cc} Y\cdot {\left[\text{min}r,\frac{W}{\gamma \left(i\right)}\right]}^{-\sigma }, & i\leq I,\\ Y{\left[\frac{W}{\gamma \left(i\right)}\right]}^{-\sigma }, & i>I. \end{array}\right. \end{array}$$
We use robots only when the cost of using the robot is less than that of labor. There exists a unique $\tilde{I}$ such that $r=W/\gamma \left(\tilde{I}\right)$. For task $\tilde{I}$, the production cost is equal for labor and capital. Let $I^{*}=\text{min}\left\{I,\tilde{I}\right\}$. All tasks are produced with capital if *i* ≤ *I*^*∗*^, while all tasks are produced with labor if *i* > *I*^*∗*^.
So, the demand for capital and labor is as follows:
  (6) $$\begin{array}{c} k\left(i\right)=\left\{\begin{array}{cc} Yr^{-\sigma }, & i\leq I^{*},\\ 0, & i>I^{*}, \end{array}\text{ and}\right.\\ l\left(i\right)=\left\{\begin{array}{cc} 0, & i\leq I^{*},\\ Y{\left(\frac{W}{\gamma \left(i\right)}\right)}^{-\sigma }, & i>I^{*}. \end{array}\right. \end{array}$$
When the capital and labor market clear, the following equations hold:
  (7) $$\begin{array}{c} \left(I^{*}-N+1\right)Y\cdot r^{-\sigma }=K,\\ Y\cdot \int_{I^{*}}^{N}{\left(\frac{W}{\gamma \left(i\right)}\right)}^{-\sigma }\text{d}i=L^{s},\\ \left(I^{*}-N+1\right)\cdot p^{1-\sigma }+\int_{I^{*}}^{N}{\left(\frac{W}{\gamma \left(i\right)}\right)}^{1-\sigma }\text{d}i=1. \end{array}$$
(II). Task-based model for robots and employment

Suppose that there are *C* regions in the economy, and each region has *I* industries. The output of each region is *Y*_*C*_, and the output of each industry in the region is *Y*_*ci*_, *c* ∈ *C*. Consumption of the region is *X*_*C*_, and the consumption of the region for each industry is *X*_*ci*_. Suppose that there is no trade, then we get the following equation:

(8) $$\begin{array}{c} Y_{C}=X_{C},\\ Y_{ci}=X_{ci},\\ Y_{C}={\left(\sum_{i\in I}v_{i}^{1/\sigma }\cdot Y_{ci}^{\sigma -1/\sigma }\right)}^{\sigma /\sigma -1}, \end{array}$$

where *v*_*i*_ is the share of industry *i*, *σ* is substitution elasticity, and ∑_*i*∈*I*_*v*_*i*_=1, the price is *p*_*ci*_^*X*^ for industry *i*'s output. Output is produced by combining capital *K* with continuous tasks *s* ∈ [0,1]. A task can be produced by robots or labor. *x*_*ci*_(*s*) shows the quantity of task *s*.

(9) $$\begin{array}{c} X_{Ci}=A_{Ci}{\left[\underset{s\in \left[0,1\right]}{\text{min}}\left\{x_{Ci}\left(s\right)\right\}\right]}^{\alpha }\cdot K_{Ci}^{1-\alpha }. \end{array}$$

According to the basic model in *I*, there is a boundary *θ*_*i*_ when tasks *s* ≤ *θ*_*i*_. Tasks can be performed using labor or robots while they must be performed using labor if tasks *s* > *θ*_*i*_.

(10) $$\begin{array}{c} X_{Ci}=\left\{\begin{array}{cc} \gamma_{M}\cdot M_{Ci}\left(s\right)+\gamma_{L}L_{Ci}\left(s\right), & s\leq \theta_{i},\\ \gamma_{i}\cdot L_{Ci}\left(s\right), & s>\theta_{i}, \end{array}\right. \end{array}$$

where *γ*_*M*_ and   *γ*_*L*_ are productivity of robot and labor, respectively. *M*_*Ci*_(*s*) and *L*_*Ci*_(*s*) are numbers of robots and labors in task *s*, respectively.

Robots are produced using investment *I*_*C*_ with *M*_*C*_=*D* · (1+*η*)*I*_*C*_^1/1+*η*^. So *Y*_*C*_=*C*_*C*_+*I*_*C*_. *C*_*C*_ is the consumption of household. Let *L*_*C*_ is the labor supply and *W*_*C*_ is wage. The rental price of robot is *R*_*C*_^*M*^ and nonrobot capital is fixed at *K*_*C*_ with price *R*_*C*_^*k*^.

We use robot only when its cost is less than that of using labor (i.e., *R*_*C*_^*M*^/*V*_*M*_ < *W*_*C*_/*γ*_*L*_). Let *π*_*C*_=1 − *R*_*C*_^*M*^/*γ*_*M*_/*W*_*C*_/*γ*_*L*_. We use robot when *π*_*C*_ > 0. Now, we look at the impact of robots on employment. We get the following equations:

(11) $$\begin{array}{c} M_{C}=D\cdot \left(1+\eta \right)\cdot I_{C}^{1/1+\eta },\\ I_{C}=D^{-1-\eta }\cdot {\left(1+\eta \right)}^{-1-\eta }\cdot M_{C}^{1+\eta },\\ C_{C}=Y_{C}-I_{C}=Y_{C}-D^{-1-\eta }\cdot {\left(1+\eta \right)}^{-1-\eta }\cdot M_{C}^{1+\eta }. \end{array}$$

In region *c*, the following equations define the first-order condition for the representative household:

(12) $$\begin{array}{c} W_{C}=BC_{C}^{\psi }\cdot L_{C}^{\varepsilon }\\ =B\cdot \left[Y_{C}-D^{-1-\eta }\cdot {\left(1+\eta \right)}^{-1-\eta }\cdot M_{C}^{1+\eta }\right]\cdot L_{C}^{\varepsilon }. \end{array}$$

For robots, we obtain the following equation:

(13) $$\begin{array}{c} R_{C}^{M}=\frac{dI_{C}}{dM_{C}},\\ R_{C}^{M}=D^{-1-\eta }\cdot {\left(1+\eta \right)}^{-1-\eta }\cdot \left(1+\eta \right)\cdot M_{C}^{\eta +1-1},\\ R_{C}^{M}=D^{-1-\eta }\cdot {\left(1+\eta \right)}^{-\eta }\cdot M_{C}^{\eta }. \end{array}$$

Price of industry *i* is marginal cost, so we get the following equation:

(14) $$\begin{array}{c} p_{Ci}^{X}=\frac{1}{A_{Ci}}\cdot {\left[\theta_{i}\frac{R_{C}^{M}}{\gamma_{M}}+\left(1-\theta_{i}\right)\frac{W_{C}}{\gamma_{L}}\right]}^{\alpha }\cdot {\left(R_{C}^{k}\right)}^{1-\alpha }. \end{array}$$

The share of labor in tasks is as follows:

(15) $$\begin{array}{c} S_{Ci}^{L}=\frac{W_{C}L_{Ci}}{\partial p_{Ci}^{X}X_{Ci}}\\ =\frac{\left(1-\theta_{i}\right)\left(W_{C}/\gamma_{L}\right)}{\theta_{i}\left(R_{C}^{M}/\gamma_{M}\right)+\left(1-\theta_{i}\right)\left(W_{s}/\gamma_{L}\right)}, \end{array}$$

*αS*_*Ci*_^*L*^ is the share of labor in the value added of industry *i*. 1 − *α* of total cost are paid to capital.

(16) $$\begin{array}{c} \sum_{i\in I}v_{i}{\left(p_{Ci}^{X}\right)}^{1-\sigma }=1. \end{array}$$

From (15), we get the wage in the region *c*: *W*_*C*_ · *L*_*Ci*_=*αS*_*Ci*_^*L*^ · *p*_*Ci*_^*X*^ · *X*_*Ci*_

(17) $$\begin{array}{c} W_{C}L_{C}=\sum_{i\in I}\alpha S_{Ci}^{L}v_{i}p_{Ci}^{X/1-\sigma }\cdot Y_{C}. \end{array}$$

Similarly, the demand for robot and capital can be represented as follows:

(18) $$\begin{array}{c} R_{C}^{M}\cdot M_{C}=\sum_{i\in I}\alpha \left(1-S_{Ci}^{L}\right)v_{i}\cdot p_{Ci}^{X}{\;}^{1-\sigma }\cdot Y_{C},\\ R_{C}K_{C}=\left(1-\alpha \right)\cdot Y_{C}. \end{array}$$

Because the added-value of industry *i* is *v*_*i*_ · *p*_*Ci*_^*X*^ ^1−*σ*^ · *Y*_*C*_ and labor share is *S*_*Ci*_^*L*^*α*, using (14) and (15) we get the following equation:

(19) $$\begin{array}{c} \sum_{i\in I}vi{\left(p_{ci}^{x}\right)}^{1-\sigma }=1. \end{array}$$

Then,

(20) $$\begin{array}{c} L_{Ci}=\left(1-\theta_{i}\right)\left(\frac{\alpha \left(1-\alpha \right)\left(1-\alpha /\alpha \right)v_{i}}{\gamma_{L}A_{Ci}^{1/\alpha }}\right)p_{Ci}^{X1-\sigma -\left(1/\alpha \right)}Y_{C}^{1/\alpha }K_{C}^{\alpha -1/\alpha }. \end{array}$$

Taking the log form as follows:

(21) $$\begin{array}{c} \ln L_{Ci}=\ln\left(1-\theta_{i}\right)+\ln\frac{\alpha {\left(1-\alpha \right)}^{1-\alpha /\alpha }v_{i}}{\gamma_{L}A_{i}^{1/\alpha }}-\left(\sigma +\frac{1}{\alpha }-1\right)\ln p_{Ci}^{X}+\frac{1}{\alpha }\ln Y_{C}+\frac{\alpha -1}{\alpha }\ln K_{C}. \end{array}$$

Differentiating both sides, we obtain the following equation:

(22) $$\begin{array}{c} d\ln L_{Ci}=-\frac{1}{1-\theta_{i}}\text{d}\theta_{i}+\frac{1}{\alpha }d\ln Y_{C}-\left(\sigma +\frac{1}{\alpha }-1\right)d\ln p_{Ci}^{X}. \end{array}$$

There are three different forces of robots that affect labor demand, as shown in (22). The first part is the displacement effect. When *θ*_*i*_ increases, it means that more robots are involved in replacing labor and this effect always reduces the labor force. The second part is a positive productivity effect. Automation reduces costs and increases productivity and labor demand among industries. Finally, workers can be transferred from the automated tasks to the nonautomated tasks, and thus they can specialize in the performance of new tasks.

### 3.2. Data Description

#### 3.2.1. Exposure to Robots

Robotics data for industries comes from the IFR (IFR, 2019). The IFR compiled annual robot use data for 50 countries from 1993 to 2019. The use of the IFR data for studying changes in employment and robot adoption has been widely reported in the literature. Using the robot data, Acemoglu and Restrepo [7], Graetz and Michaels [6], Dauth et al. [9], Carbonero et al. [34], and Chiacchio et al. [8] have explored the impact of robot adoption on employment in the United States, Germany, and different EU countries [7–9, 34]. We use data from the period between 2010 and 2019 since robots in China have been growing rapidly since the early 2000s.  Table 1 lists 13 industries for which we collected robot data. Similar to Du and Lin [14], we compare these industries with those in the Chinese national standard (GB/T 4754–2017). In this industry classification standard, there are 13 industries, as shown in column 3 of Table 1. The first column of Table 1 represents the industries with robot data. The second column is the industry label, such as information technology (IT) and scientific research and technical (R&T) services. More robots are utilizing the IFR (IFR, 2019) data than ever. The number of robots currently utilized was measured by the operational stock of robots.

China's use of industrial robots began in the early 1970s but developed slowly thanks to abundant labor resources and backward technology. The popularity of robots began to increase in the mid-1980s during the period of reform and opening up. Robots were listed as the key national scientific research program in the seventh five-year plan. When the National High Technology Research and Development Program (“863” Program) of China was launched, the theme of intelligent robots was created, and robotics was listed as a crucial field in the “Made in China 2025” document.

**Figure 1:**
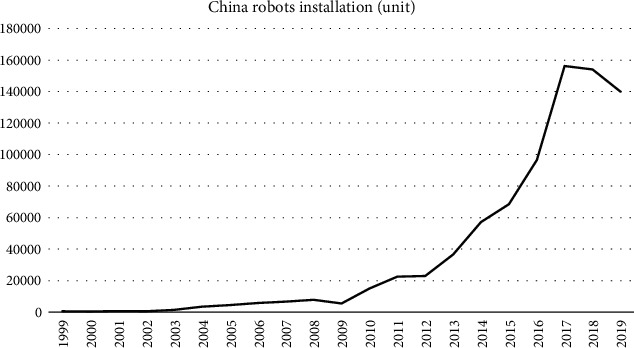
China's robots installations from 1999 to 2019. (Source: IFR, 2019).

From the robot operation data for the most recent ten years, it is evident that the robot operations in China have been in a state of continuous growth. At the same time, the United States, Germany, Japan, and South Korea have experienced zero growth or even negative change in the rate of robot ownership in the past ten years. Since 2013, China has been the world's largest industrial robot market.  Figure 1 shows robot installations in China between 1999 and 2019 measured as the number of units. The number of robot installations was about 550 units in 1990. After ten years of slow growth, robot installations increased from 5525 units in 2009 to 156,000 in 2017. In 2017 and 2018, China's industrial robot installations accounted for 38% of the world's total. In 2019, a total of 140,000 units were installed, which is 9% less than that in 2018 but still greater than the total number of robots installed in Europe and the United States.

#### 3.2.2. Labor Market Data

**Figure 2:**
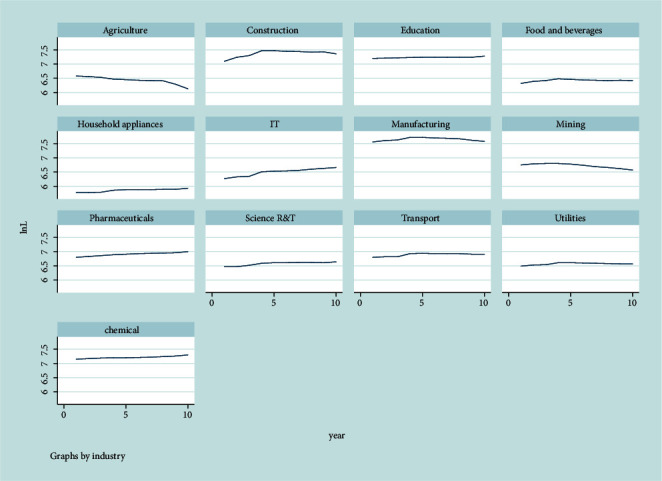
Employed persons by industry from 2000 to 2019. Notes: The results were graphed using Stata 15. This figure displays the industrial data to identify the trend of the change in the number of people employed from 2010 to 2019.

The China Statistical Yearbook compiled by the National Bureau of Statistics of China covers the employment numbers for each industry from 2010 to 2019.  Figure 2 displays the industrial data to identify the trend of the change in the number of people employed from 2010 to 2019.

We divide industries into four categories based on sectors and employment trends. The first group (A) is the industries where employment has tended to rise, including IT, pharmaceuticals, science R&T, and chemicals. The second group (B) includes agriculture and mining. Their employment has followed a downward trend for the last ten years. The third group (C) is the rest of the tertiary sector, including education, food and beverages, utilities, household appliances, and transportation. In these industries, employment has remained unchanged over the decade of the study period. The fourth group (D) is the industries in the secondary sector, including construction and manufacturing, with a growing trend of employment until 2013 but decline since 2014. The first is the industries where employment is rising, including IT, pharmaceuticals, science R&T, and chemicals. Based on Table 2, these four industries require a relatively high level of education and about 70% of employees have a college degree or above. For example, the proportion of college, university, and graduate and higher level attainment of urban employed persons in the IT industry in 2019 was 27.5, 39.4, and 4.7, respectively.

#### 3.2.3. Control Variables

Different theories exist in the literature regarding the effect of technological progress on employment, among them classical theory, Marxist theory, neoclassical theory, new growth theory, Schumpeter's innovation theory, and business cycle theory. Up to now, several studies have revealed a correlation between technological progress and employment [19–21, 35–38]. Brouwer et al. [35] conducted two innovative studies in the Netherlands to estimate the effects of technological progress on employment. They found a positive effect caused by product-related R&D activities but an adverse effect in relation to overall R&D investments [35]. Greenan and Guellec [39] used market research in France from 1991 to analyze employment growth from 1986 to 1990. They found positive effects for both process and product innovation, with more muted effects for process innovations [39]. Bloom et al. estimated that, thanks to progress in artificial intelligence, more than 700 million new jobs will be created between 2010 and 2030 globally [19]. Acemoglu and Restrepo believe that, from the perspective of the history of science and technology, while rendering certain jobs obsolete, technological innovation will create many more new jobs in the long-run. The compensation effect produced by the new positions can offset the substitution effect [20]. Gregory et al. studied data from 27 European countries during the period from 1999 to 2010. They found that conventional substitution resulting from technological change led capital to replace labor in production, thereby eliminating about 9.6 million jobs. In comparison, the spillover effect from product demand brought about by technological progress led to an increase of nearly 21 million jobs. On the whole, technological progress has had a positive impact on employment levels for the European labor force [21].

Expenditure on R&D, contractual value deals in domestic technical markets (CVD), foreign direct investment (FDI), imports of capital goods, technology purchases, patent citations, and the index of industrial robots are some of the most commonly used indicators of technological change. Interestingly, all of these indicators catch different dimensions of technology. This study uses expenditure on R&D activities and FDI to model technological progress. The amounts of FDI and R&D for each industry were obtained from 2010 to 2019 from the China Statistical Yearbook.

Since the new millennium, China's R&D expenditure has increased at an average annual rate of about 20%. R&D expenditure has expanded from 89.6 to 2443 billion Yuan in 2000 and 2020, respectively, accounting for 0.89% to 2.40% of GDP. Especially eight years ago, China became the world's second-largest consumer, which is a major phenomenon in terms of its global economic status and deserves attention. From 2000 to 2016, China's contribution to the worldwide R&D economic expansion was 27.4%, close to the 29.5% growth of the United States.

### 3.3. The Empirical Model

According to our discussion in the theoretical framework, there are three forces about the power of robots affecting employment, including displacement, productivity, and composition effects [7]. To explore the total impact of robots on employment in different industries, we can posit the following formula:

(23) $$\begin{array}{c} \ln L_{it}=\alpha_{0}+\alpha_{1}\ln\text{ Robot}{\text{Exposure}}_{it}+x_{it}^{L}+\varepsilon_{it}, \end{array}$$

where *L*_*it*_ is the level of employment; robot exposure refers to the penetration of industrial robots in industry *i* in year *t*, which is equal to the stock of industrial robots in Chinese industry *i* in *t* years divided by the employment level of *i* industry in China in year *t*; and *x*_*it*_^*L*^ represents other factors affecting labor demand. In our empirical model, we use R&D and FDI as control variables. To standardize the data, we took log form for all the variables.

The correlation between robot exposure and employment was tested using panel data, which provides richer models and estimation methods than cross-sectional data. An extreme strategy for estimating panel data is pooled regression as cross-section data, requiring each individual in the sample to have exactly the same regression equation. The disadvantage of pooled regression is that it ignores the heterogeneity of individuals, which may relate to explanatory variables and lead to inconsistent estimates.

In practice, a compromise estimation strategy is often adopted; it is assumed that individual regression equations have the same slopes but different intercepts to capture heterogeneity. This model is called the individual-specific effects model and is formulated as below.

(24) $$\begin{array}{c} y_{it}=x_{it}^{\prime }\beta +z_{i}^{\prime }\delta +u_{i}+\varepsilon_{it}\left(i=1,\ldots ,n;t=1,\ldots ,T\right),\\ L^{D}=F\left(W,Q^{D},T\right), \end{array}$$

where *z*_*i*_ is a time-invariant individual characteristic. *x*_*it*_ can vary with individuals and over time. The disturbance term consists of *u*_*i*_+*ε*_*it*_, called composite error term. Among them, the unobserved random variable *u*_*i*_ is the intercept term representing individual heterogeneity, namely individual effects. *ε*_*it*_ is a disturbance term that varies with individual and time, known as idiosyncratic error.

If *u*_*i*_ relates to an explanatory variable, it signals the fixed effects (FE) model. If *u*_*i*_ is not correlated with all explanatory variables (*x*_*it*_, *z*_*i*_), it implies the random effects (RE) model. Hence, this research use the Hausman check if this model follows FE or RE model.

## 4. Results

### 4.1. The Empirical Results

Table 3 shows the descriptive statistics of the relevant variables used in this paper. These statistics are the mean, maximum, minimum, standard deviation, and number of observations. For example, the minimum, maximum, mean, and standard deviation of the log form of employment in group A are 2.28, 3.30, 2.80, and 0.30, respectively. *T* is years, *n* represents four industries in the first group, and the number of observations is 40.  Table 4 shows the statistical results for the above groups. There is a significant positive correlation between robots' exposure and labor demand for Group A. These results show that the use of robots promotes high-skilled employment. A 10% increase in robot density leads to a 1.1% rise in high-tech jobs. The Hausman test statistics reject the null hypothesis of the RE model and accept the FE model at a 5% statistically significant level. Also, the resulted F statistics reject the pool regression and accept the FE model.

Group B shows a significant negative correlation between robots' exposure and labor demand. According to the Hausman test and F test, the FE model is superior to the RE and pooled regression model. Another finding was that R&D promotes employment in traditional industries according to the FE model, which explains the fact that various technological transformations or upgrades have a corresponding effect on workers. China is predominantly agricultural and very populous. According to the National Bureau of Statistics, employment in primary industries, mainly agriculture, accounted for 50% of the total workforce in 2001, 37% in 2010, and only 24% in 2020.

The proportion of the population employed in primary industry has declined year by year. Of the country's more than 600 million farmers, very few are engaged in the agricultural sector. More people have chosen to enter secondary and tertiary industries to go elsewhere for work or to start a small business. With the rapid development and industrialization of China, the R&D of agricultural robots has been gradually expanding. Agricultural robots can be engaged in planting, spraying pesticides, harvesting, and other field operations, and they can play an essential role in animal husbandry. This not only saves human resource costs but also improves quality control ability and enhances resilience. For the mining industry, there are many problems with underground production operations in coal mines: high accident rates, harsh operating conditions, serious environmental pollution, and high disaster risk. Faced with high-risk underground operations, robots have become an essential means of achieve the safe and efficient production goals in coal mines. To achieve safety of coal miners, it is a general trend for robots to replace miners in underground operations. Coal mine production will therefore develop toward the use of unmanned, autonomous, intelligent, and highly efficient robots in the future. Artificial intelligence technology can play an irreplaceable role in that and diversified artificial intelligence technology can be applied to coal mine robots. Although the current application of artificial intelligence in the field of industrial coal mining is still in a period of exploration, with the increasing application of artificial intelligence technology in the field of coal mining, it is imperative to build unmanned mines.

For Group C, after the Hausman test and F test, the FE model is superior to the RE model and pooled regression.  Table 4 indicates that the use of robots has a positive effect on the third employment sector. Robots have been used in the service industry for decades. Helpmate, a service robot created in the 1980s by Joseph F·Engelberger, the so-called “father of robotics,” delivers meals, medicine, and supplies to hospital patients [16]. Helpmate, of course, is a square box, impersonal and rudimentary. Service robots are growing fast in Japan in another direction: entertainment robots. In recent years, robots have been increasingly used in the service industry. As Engelberger predicted, robots are more likely to be used in the service industries, including maintenance, repair, transportation, cleaning, security, rescue, and domestic tasks and nursing [16]. In recent years, modern information technologies have developed rapidly including cloud computing, the “Internet of Things,” artificial intelligence, and “big data.” A series of proactive policies continued to develop, including the transformation of the real economy, cross-border e-commerce, and support for rural e-commerce. Also, new forms of online retail and orders have risen, creating a large number of new jobs in wholesale and retail trade, hotels, and restaurant industries.

In Group D, the results of the Hausman and LR tests in MLE support the RE model against the FE and pooled regression models. Nonetheless, the LM test results suggest a pooled regression against the RE model. In this group, there is no significant correlation between robot density and employment in the secondary sector. Secondary industries involve many industries, such as steel manufacturing, automobile production, and wired and wireless communication. Some companies have tried to liberate front-line workers by using industrial robots in recent years. Foxconn, the largest mobile phone manufacturer, uses industrial robots to perform the tasks previously performed by workers on production lines. Now, some domestic manufacturing enterprises have begun to replace front-line operators with industrial robots, improving their efficiency.

### 4.2. Robustness Check

Table 5 shows the results of different estimated regression methods on the employment of robots. The regression results of FE using clustering robust standards in Table 5 show that robots have a positive relationship with the employment of the first and third groups, and a negative relationship with the employment of the second group. Both of them are significantly valid at 0.05, which indicates that the use of industrial robots increases employment in tertiary sectors and high-tech industries, but is ineffective in employment in agriculture and mining industries. Table 5 shows that the results obtained by different regression estimation methods (i.e., FE_Robust and LSDV) are the same, which verifies the validity and robustness of the basic FE regression and the empirical results.

In addition to using different regression methods, we add a lagged core explanatory variable for endogeneity problems. In this paper, the lag variable L.lnRobot of explanatory variable lnRobot is used as a substitute variable to conduct regression tests.  Table 6 shows the results. The coefficients of lag variable L.lnRobot on the employment of the three groups are 0.096, -0.202, and 0.033, respectively, and significant at the 1% level, indicating that the regression results are still robust after considering endogeneity.

Following [40], the equation of demand for labor is as follows:

(25) $$\begin{array}{c} L^{D}=F\left(W,Q^{D},T\right), \end{array}$$

where *L*^*D*^ is labor demand or the desired level of employment, *W* is the wage rate, *Q*^*D*^ is the output or product demand, and *T* is the technology. We add wage and value-added (VA) in our model, and the robot, R&D, and FDI represent technology.  Table 7 displays that industrial robot still has a significant impact on employment under the model of increasing control variables, indicating that the regression results are still robust after the intervention of increasing control variables.

### 4.3. Discussion of the Results

According to the capital–skill complementarity hypothesis discussed in the literature review, technological progress leads to an increase in demand for skilled labor and a decrease in demand for unskilled labor. An initial objective of this study was to identify the relationship between industrial robot adoption and China's employment in different industries and skills. Our results suggested that an association existed between robot adoption and employment across industries. There is a significant positive correlation between robots' exposure and labor demand for high-skilled employment and third sector employment. A 10% increase in robot density leads to a 1.1% rise in high-tech jobs. However, multiple regression analysis reveals that robots have reduced employment in traditional industries such as agriculture and mining. This study supports evidence from previous observations [11, 12, 41, 42] that industrial robots are a skilled-biased technology change.

Using data about Germany, Dauth et al. found that industrial robots reduce manufacturing employment, but the reduced employment is offset by increased employment in the tertiary industry [9]. The industry-based estimates in this study are inconsistent with those of Acemoglu and Restrepo [7], which are based on data about the United States. Their findings showed that the negative effects of robot adoption on employment are mainly felt in highly mechanized industries, such as automobile manufacturing, chemical, pharmaceutical, and food manufacturing. Robotic applications will promote labor employment in industries such as finance, the public sector, and nonrobotized manufacturing [7]. These results are inconsistent for two reasons. First, compared with developed countries such as the United States, China's robot adoption is still in its early stages. The first adopters of robotic production technology can use this competitive advantage to expand their market share, thereby increasing the demand for labor. This role is particularly prominent in the capital- or technology-intensive industries, such as information transmission, software and information technology, communication equipment, and computer and other electronic equipment manufacturing. Second, with the rise in labor costs in China, labor-intensive industries such as construction, manufacturing, and mining face more prominent cost pressures, indicating that such industries have more substantial economic incentives to replace labor with robots.

## 5. Conclusion

Our study aims to assess how robots contribute to the employment of different industries and skills. The results of this investigation show a significant positive correlation between robots' exposure and labor demand for the third sector of employment. The results show that the use of robots promotes high-skilled talent employment. However, multiple regression analysis reveals that the use of robots has reduced employment in traditional industries such as agriculture and mining. This study represents the first comprehensive assessment of how industrial robots contribute to the employment of different industries and skills of China. A limitation of this study is that the robot data is only from 2010, because the use of robots in China has started late but has increased rapidly since the 2000s.The use of robots affects not only employment but also other aspects of the labor market and the economy as a whole. There is still uncertainty about whether robot technical progress impacts wage inequality. Further work needs to be done to establish whether the application of robots is conducive to improving the labor share of national income. Much uncertainty still exists about the relationship between the industry robots, total factor productivity, and added-value of China. Further research needs to examine more closely the links between industrial robots and productivity.

The robot industry will be the main engine of economic growth in the next few years. The progress of robotics is inevitable. There will be unprecedented significant development and improvement in the field of robotics. We must accept this trend and seize this opportunity to leap into economic development. Obviously, under the state's leadership, robot technology as a whole is helping to promote positive economic growth, and it has become an opportunity for people to find jobs again. The findings of this study have a number of important implications for future practice. Studying the impact of robots on economic outcomes may provide guidelines for governments about China's economic growth and employment in the future.

This study showed that the employment promotion effect of robot adoption mainly lay in the middle- and high-skilled labor groups, indicating that intelligent manufacturing provided by robots needs a large medium- and high-skilled labor force to match it. The average skill level in China's current labor force was low, and the low-skill labor force accounted for a large proportion of the total labor force. Therefore, China should further strengthen the guidance and expenditure on vocational skill education and training, provide abundant labor resources for intelligent manufacturing, and reduce frictional unemployment caused by technological change.

We also found the employment substitution effect of robot adoption in traditional industries, such as agriculture and mining. The government should provide more guidance and support for unemployment insurance, job-transfer training, and especially new skills learning. The government should also alleviate the employment impact of new technology adoption on traditional industries and ensure a fuller and higher employment quality.

We provide three recommendations for the education system. First, higher vocational education should be developed. The state can establish and improve the majors of artificial intelligence and robotics, train high-skilled professors, and establish practice bases to cultivate intelligent, automated, and information-based technical workers. Second, re-employment training should be strengthened. In addition to the existing higher vocational colleges, the government should support the establishment of more re-employment training centers. Subsidies should be increased for the return of low-skilled young people to advance their studies and for the unemployed to undergo re-employment training; this would reduce re-education costs for both groups and enable them to acquire competent skills in intelligent, automated, and information-based production positions. Finally, relevant preferential policies should be introduced to guide the flow and transfer of labor among various industries of the national economy in an orderly manner so that citizens do not need to bear more costs for acquiring new labor skills and positions.

## Figures and Tables

**Table 1:** List of all industries based on the China National Standard (GB/T 4754-2017).

Included in robotics data	Label	Code description
√	Agriculture	Agriculture, forestry, animal husbandry and fishery
√	Mining	Mining
√	Manufacturing	Manufacturing
√	Utilities	Production and supply of electricity, heat, gas and water
√	Construction	Construction
√	Transport	Transport, storage and post
√	IT	Information transmission, software and information technology
√	Food and beverages	Hotels and catering services
√	Science R&T	Scientific research and technical services
√	Chemical	Management of water conservancy, environment
√	Household appliances	Services to households, repair and other services
√	Education	Education
√	Pharmaceuticals	Health and social service

*Notes*.  Table 1 lists 13 industries for which we collected robot data from IFR. We compared these with the industries in the Chinese national standard (GB/T 4754-2017) as shown in column 3 of Table 1. The first column of Table 1 represents the industries with robot data. The second column is the industry label, such as information technology (IT) and scientific research and technical (R&T) services.

**Table 2:** Educational attainment of urban employed persons by industry in 2019.

Industries	Total	Junior secondary school and below	Senior secondary school	Medium vocational education	High vocational education	College	University	Graduate and higher level
Agriculture	100	89.4	6.6	1.5	0.2	1.5	0.7	0.0
Mining	100	36.4	21.7	12.0	2.6	16.2	10.5	0.7
Manufacturing	100	47.4	18.4	9.4	1.7	13.3	9.0	0.8
Utilities	100	20.4	18.3	10.8	2.5	24.4	21.6	2.1
Construction	100	61.9	14.7	4.6	0.9	10.0	7.4	0.4
Transport	100	46.3	21.2	8.4	1.9	13.5	8.3	0.3
IT	100	8.8	10.7	6.7	2.0	27.5	39.4	4.7
Science R&T	100	9.9	10.2	5.2	2.1	23.0	38.9	10.7
Chemical	100	44.6	14.5	6.5	1.7	16.2	15.1	1.3
Households	100	53.6	19.7	7.8	1.8	10.8	6.0	0.2
Education	100	8.8	6.6	5.8	1.9	23.7	45.0	8.3
Pharmaceuticals	100	10.9	8.0	10.6	2.3	30.2	33.4	4.5

*Source*. China Population Statistics Yearbook (2019).

**Table 3:** Descriptive statistics.

Group	Variables	Min	Max	Mean	Std. dev	Observations		
Group A	lnL	2.28	3.30	2.80	0.30	*N* = 40	*n* = 4	*T* = 10
	lnRobot	−1.54	2.60	0.70	0.87	*N* = 40	*n* = 4	*T* = 10
	lnFDI	3.81	6.32	5.07	0.71	*N* = 40	*n* = 4	*T* = 10
	lnRD	4.91	7.18	5.77	0.72	*N* = 40	*n* = 4	*T* = 10

Group B	lnL	2.13	2.80	2.57	0.18	*N* = 20	*n* = 2	*T* = 10
	lnRobot	−1.79	0.48	-0.94	0.69	*N* = 20	*n* = 2	*T* = 10
	lnFDI	3.98	5.34	4.96	0.36	*N* = 20	*n* = 2	*T* = 10
	lnRD	3.90	6.32	5.18	0.99	*N* = 20	*n* = 2	*T* = 10

Group C	lnL	1.78	3.28	2.59	0.47	*N* = 50	*n* = 5	*T* = 10
	lnRobot	−1.51	2.19	0.58	1.09	*N* = 50	*n* = 5	*T* = 10
	lnFDI	2.60	5.75	4.85	0.78	*N* = 50	*n* = 5	*T* = 10
	lnRD	2.92	4.88	4.11	0.60	*N* = 50	*n* = 5	*T* = 10

Group D	lnL	3.10	3.72	3.51	0.17	*N* = 20	*n* = 2	*T* = 10
	lnRobot	−1.89	2.25	0.31	1.44	*N* = 20	*n* = 2	*T* = 10
	lnFDI	4.96	6.72	5.89	0.76	*N* = 20	*n* = 2	*T* = 10
	lnRD	4.17	7.21	5.37	1.17	*N* = 20	*n* = 2	*T* = 10

**Table 4:** Effects of robots on employment of the four group.

	Group A			Group B			Group C			Group D		
	(1) OLS	(2) FE	(3) RE	(1) OLS	(2) FE	(3) RE	(1) OLS	(2) FE	(3) RE	(1) OLS	(2) FE	(3) RE
lnRobot	0.258^*∗∗*^	0.108^*∗∗∗*^	0.258^*∗∗∗*^	−0.177	−0.194^*∗∗∗*^	−0.177	0.039	0.047^*∗∗∗*^	0.045^*∗∗∗*^	0.121	0.137^*∗∗*^	0.121^*∗∗*^
	(3.76)	(13.28)	(7.66)	(−4.54)	(−8.55)	(−6.46)	(1.53)	(6.49)	(2.62)	(0.69)	(2.63)	(2.35)
lnFDI	−0.402^*∗∗*^	−0.032	−0.402^*∗∗∗*^	−0.047	0.021	−0.047	0.311^*∗∗∗*^	−0.018	−0.072^*∗*^	−0.006	0.190	−0.006
	(−4.78)	(−1.38)	(−9.75)	(−0.95)	(0.49)	(−1.04)	(−7.39)	(−0.99)	(−1.79)	(−0.03)	(1.12)	(−0.08)
lnRD	0.057	0.029	0.057	−0.065	0.172^*∗∗*^	−0.065^*∗∗∗*^	0.641^*∗∗∗*^	0.018	0.084^*∗∗*^	−0.014	−0.004	−0.014
	(1.72)	(0.92)	(1.54)	(−2.17)	(2.21)	(−3.07)	(5.19)	(1.14)	(2.24)	(−0.15)	(−0.10)	(−0.35)
_Cons	4.331^*∗∗∗*^	2.717^*∗∗∗*^	4.331^*∗∗∗*^	2.979^*∗∗*^	1.401^*∗∗*^	2.979^*∗∗∗*^	1.460^*∗∗*^	2.592^*∗∗∗*^	2.572^*∗∗∗*^	3.579	2.368^*∗∗*^	3.579^*∗∗∗*^
	(8.02)	(12.12)	(17.28)	(50.32))	(2.64)	(14.54)	(2.90)	(27.49)	(11.36)	(2.29)	(2.22)	(7.28)
*N*	40	40	40	20	20	20	49	49	49	20	20	20
*R* ^2^	0.73	0.901		0.890	0.792		0.793	0.427		0.782	0.276	
*F*	.	119.941			25.39		51.884	14.268		.	3.752	

*Notes*. This table reports the impact of industrial robots on employment of the four groups. The dependent variable is employment at the industrial level. All other variables are defined in Section 3.2. OLS is the pooled regression and FE, RE are the fixed and random effect model, respectively. t statistics are reported in parentheses. ^*∗∗∗*^, ^*∗∗*^, and ^*∗*^ indicate significance at the 1%, 5%, and 10% levels, respectively.

**Table 5:** Result of regression analysis of different estimation methods of the four groups.

	Group A		Group B		Group C	
	(4) FE_Robust	(5) LSDV	(4) FE_Robust	(5) LSDV	(4) FE_Robust	(5) LSDV
lnRobot	0.108^*∗∗∗*^	0.108^*∗∗∗*^	0.194^*∗∗*^	−0.194^*∗∗*^	0.047^*∗∗∗*^	0.047^*∗∗∗*^
	(13.93)	(13.34)	(−20.59)	(−19.94)	(9.34)	(8.92)
lnFDI	−0.032	−0.032	0.021	0.021	−0.018	−0.018
	(−1.07)	(−1.03)	(0.29)	(0.28)	(−1.56)	(−1.49)
lnRD	0.029	0.029	0.172	0.172	0.018	0.018
	(0.76)	(0.73)	(4.18)	(4.04)	(1.06)	(1.01)
_Cons	2.717^*∗∗∗*^	3.082^*∗∗∗*^	1.401	1.166	2.592^*∗∗∗*^	3.237^*∗∗∗*^
	(12.01)	(13.82)	(2.51)	(1.84)	(31.15)	(40.29)
*N*	40	40	20	20	49	49
*R* ^2^	0.909	0.993	0.805	0.929	0.478	0.996
*F*	2.1*e* + 04	.	.	.	269.906	.

*Notes*. This table reports the results of the robustness test in Section 4.2. FE_Robust is FE using clustering robust standard and LSDV is least-squares dummy variables regression. The dependent variable is employment at the industrial level. All other variables are defined in Section 3.2. t statistics are reported in parentheses. ^*∗∗∗*^, ^*∗∗*^, and ^*∗*^ indicate significance at the 1%, 5%, and 10% levels, respectively.

**Table 6:** Adding lagged core explanatory variables to deal with endogeneity.

	Group A	Group B	Group C
	(6) lnL	(6) lnL	(6) lnL
L.lnRobot	0.096^*∗∗∗*^	−0.202^*∗∗∗*^	0.033^*∗∗∗*^
	(12.10)	(−7.83)	(4.45)
lnFDI	−0.028	0.015	−0.015
	(−1.24)	(0.35)	(−0.85)
lnRD	0.060^*∗*^	0.180^*∗*^	0.035^*∗∗*^
	(1.91)	(1.95)	(2.29)
_Cons	2.552^*∗∗∗*^	1.340^*∗∗*^	2.535^*∗∗∗*^
	(12.56)	(2.16)	(25.97)
*N*	36	18	44
*R* ^2^	0.887	0.774	0.293
*F*	93.396	20.750	8.265

*Notes*. This table reports the results of the robustness test in Section 4.2. The lag variable L.lnRobot of explanatory variable lnRobot is used as a substitute variable to conduct regression test. The dependent variable is employment at the industrial level. All other variables are defined in Section 3.2. t statistics are reported in parentheses. ^*∗∗∗*^, ^*∗∗*^, and ^*∗*^ indicate significance at the 1%, 5%, and 10% levels, respectively.

**Table 7:** Result of FE model by adding control variables for intervention.

	Group A	Group B	Group C
	(7) lnL	(7) lnL	(7) lnL
lnRobot	0.0876^*∗∗∗*^	−0.160^*∗*^	0.035^*∗*^
	(8.00)	(−2.05)	(1.78)
lnFDI	−0.0306	0.040	−0.026
	(−1.37)	(0.69)	(−1.26)
lnRD	−0.00840	0.199^*∗*^	0.016
	(−0.26)	(2.00)	(1.06)
lnWage	−0.132	−0.142	0.531^*∗∗*^
	(−0.56)	(−0.36)	(2.68)
lnVA	0.204	−0.121	−0.409^*∗∗*^
	(1.29)	(−0.40)	(−2.08)
_Cons	2.766^*∗∗∗*^	2.399	1.858^*∗∗∗*^
	(5.14)	(1.21)	(3.92)
*N*	40	20	49
*R* ^2^	0.916	0.765	0.493
*F*	86.743	13.561	11.119

*Notes*. This table reports the results of the robustness test in Section 4.2. The dependent variable is employment at the industrial level. We add wage and value-added (VA) in our model as control variables. All other variables are defined in Section 3.2. t statistics are reported in parentheses. ^*∗∗∗*^, ^*∗∗*^, and ^*∗*^ indicate significance at the 1%, 5%, and 10% levels, respectively.

## Data Availability

All variables used in this study are listed with the sources in the following: Industrial Robot: robotics data for industries comes from the International Federation of Robotics (2019). Source: https://ifr.org/worldrobotics/. Employment: the number of employed persons in urban units by sector. Source: China Statistical Yearbook compiled by the National Bureau of Statistics of China. https://data.stats.gov.cn/english/. Education: educational attainment of urban employed persons by sector and sex. Source: China population and employment Statistics yearbook complied by Department of Population and Employment National Bureau of Statistics of China. https://www.yearbookchina.com/. FDI: Foreign Direct Investment by sector, investment actually utilized. Source: China Statistical Yearbook compiled by the National Bureau of Statistics of China. https://data.stats.gov.cn/english/. R& D: intramural expenditure on R& D of R&D institutions by industrial sector in which the R&D institutions served. Source: China Statistical Yearbook on Science and Technology. https://www.yearbookchina.com/. Wage: average Wage of Employed Persons in Urban Units by _Sector. Source: China Statistical Yearbook compiled by the National Bureau of Statistics of China. https://data.stats.gov.cn/english/. Value-added: Value-added by Sector. Source: China Statistical Yearbook compiled by the National Bureau of Statistics of China. http://www.stats.gov.cn/tjsj/ndsj/2019/indexeh.htm.
